# The Methyl Ester of 2-Cyano-3,12-Dioxooleana-1,9-Dien-28-Oic Acid Reduces Endometrial Lesions Development by Modulating the NFkB and Nrf2 Pathways

**DOI:** 10.3390/ijms22083991

**Published:** 2021-04-13

**Authors:** Rosalba Siracusa, Ramona D’Amico, Marika Cordaro, Alessio Filippo Peritore, Tiziana Genovese, Enrico Gugliandolo, Rosalia Crupi, Daniela Impellizzeri, Salvatore Cuzzocrea, Roberta Fusco, Rosanna Di Paola

**Affiliations:** 1 Department of Chemical, Biological, Pharmaceutical and Environmental Sciences, University of Messina, 98166 Messina, Italy; rsiracusa@unime.it (R.S.); rdamico@unime.it (R.D.); aperitore@unime.it (A.F.P.); tgenovese@unime.it (T.G.); rfusco@unime.it (R.F.); dipaolar@unime.it (R.D.P.); 2 Department of Biomedical, Dental and Morphological and Functional Imaging, University of Messina, Via Consolare Valeria, 98125 Messina, Italy; cordarom@unime.it; 3 Department of Veterinary Sciences, University of Messina, 98168 Messina, Italy; egugliandolo@unime.it (E.G.); rcrupi@unime.it (R.C.)

**Keywords:** endometriosis, inflammation, oxidative stress

## Abstract

Endometriosis is a common gynecological disease. Here, we aimed to investigate the anti-fibrotic, anti-inflammatory, and anti-oxidative role of the methyl ester of 2-cyano-3,12-dioxooleana-1,9-dien-28-oic acid (CDDO-Me) on endometriosis. An endometriosis rat model was constructed by intraperitoneally injecting recipient rats with an equivalent of tissue from the uterus of a donor animal. Endometriosis was allowed to develop for seven days. CDDO-Me was administered on the 7th day and for the next 7 days. On day 14, rats were sacrificed, and peritoneal fluid and endometriotic implants were collected. CDDO-Me displayed antioxidant activity by activating the Nfr2 pathway and the expression of antioxidant mediators such as NQO-1 and HO-1. Moreover, it reduced lipid peroxidation and increased glutathione (GSH) levels and superoxide dismutase (SOD) activity. CDDO-Me also showed anti-inflammatory activity by decreasing the expression of pro-inflammatory cytokines in peritoneal fluids and NFkB activation. It, in turn, reduced cyclooxygenase-2 (COX-2) expression in the endometriotic loci and prostaglandin E2 (PGE2) levels in the peritoneal fluids, leading to increased apoptosis and reduced angiogenesis. The reduced oxidative stress and pro-inflammatory microenvironment decreased implants diameter, area, and volume. In particular, CDDO-Me administration reduced the histopathological signs of endometriosis and inflammatory cells recruitment into the lesions, as shown by toluidine blue staining and myeloperoxidase (MPO) activity. CDDO-Me strongly suppressed α-SMA and fibronectin expression and collagen deposition, reducing endometriosis-associated fibrosis. In conclusion, CDDO-Me treatment resulted in a coordinated and effective suppression of endometriosis by modulating the Nrf2 and NFkB pathways.

## 1. Introduction

Endometriosis is a chronic pathology characterized by endometrial-like tissue located outside the uterine cavity [1]. It is associated with impaired fecundity and chronic pelvic pain [2]. The implantation and growth of endometrial cells influence the pelvic environment, damage ovarian tissues and immune function, and reduce follicular growth, leading to infertility, dysmenorrhea, and menstrual disorder [3]. The first-line therapy for endometriosis treatment suggests oral contraceptive pills or nonsteroidal anti-inflammatory drugs [4]. However, with these therapeutic approaches, many patients still display pelvic pain and lesion size increase. The mechanism underling endometriosis occurrence is not fully understood, but several studies demonstrated that it is associated with disorder of the oxidative balance and the immune system [5,6].

In particular, an altered expression of antioxidant and pro-oxidant enzymes has been detected in endometrial lesions [7]. In endometriotic cells, higher levels of superoxide anions, hydrogen peroxide, and malondialdehyde (MDA), a lipid peroxides index, were observed as compared to a healthy control, while catalase concentrations and superoxide dismutase activity were downregulated in endometriotic cells [8]. All these pieces of evidence suggest a declined antioxidant activity in endometriosis patients. Redox-sensitive nuclear factor erythroid 2-related (Nrf2) is involved in the transcription of endogenous antioxidant enzymes, and its activation could protect against the oxidative stress involved in endometriosis [9].

Investigations have shown that hallmarks of endometriosis include inflammation and cell apoptosis [10]. It correlates with increased numbers of mast cells and elevations of inflammatory cytokines including interleukin (IL)-1β, IL-6, IL-2 and tumor necrosis factor (TNF)-α [11,12]. In the peritoneal fluid, these excessive levels of pro-inflammatory cytokines are regulated by the nuclear factor kappa-light-chain-enhancer of activated B cells (NFkB) pathway that leads to a proinflammatory environment supporting the growth of endometriotic lesions [13]. In this inflammatory microenvironment, high reactive oxygen species (ROS) production manages NFkB expression and other genes encoding growth and angiogenic factors [14]. In particular, endometriosis is associated with increased cyclooxygenase-2 (COX2) expression and prostaglandin E2 (PGE2) levels, apoptosis, and pathological angiogenesis. PGE2 is involved in elevating anti-apoptotic and reducing pro-apoptotic proteins. Moreover, the COX-2–PGE2–pAKT axis, perturbated during endometriosis, leads to increased expression of MMP-2 and VEGF, involved in cellular migration, invasion, and angiogenesis [15]. Many compounds naturally synthetized in plants have been employed for disease management in Asian medicine [16,17]. In recent years, to increase their usefulness, synthetic derivatives of these molecules have been developed. The methyl ester of 2-cyano-3,12-dioxooleana-1,9-dien-28-oic acid (CDDO-Me) (SigmaAldrich 218600534, Milan, Italy) has been originally developed for the treatment and prevention of cancer and inflammation [18,19]. It was described that CDDO-Me pharmacological effects are mediated by the interaction with cellular nucleophiles such as redox-sensitive sulfhydryl groups of proteins [20,21]. CDDO-Me behaves as an inhibitor of the NFkB and an activator of the Nrf2 pathways. Direct targets of CDDO-Me are IkB kinase (IKKβ) and Kelch-like ECH-associated protein 1 (Keap1). In fact, its molecular structure is similar to that of cyclopentenone prostaglandins, which play an important role in the resolution of inflammation, activating Nrf2 and suppressing NFkB activity [22,23].

The aim of this study was the investigation of the effects of CDDO-Me on oxidative stress and inflammation induced by endometriosis.

## 2. Results

### 2.1. Effect of CDDO-Me Treatment on Oxidative Stress

**Figure 1:**
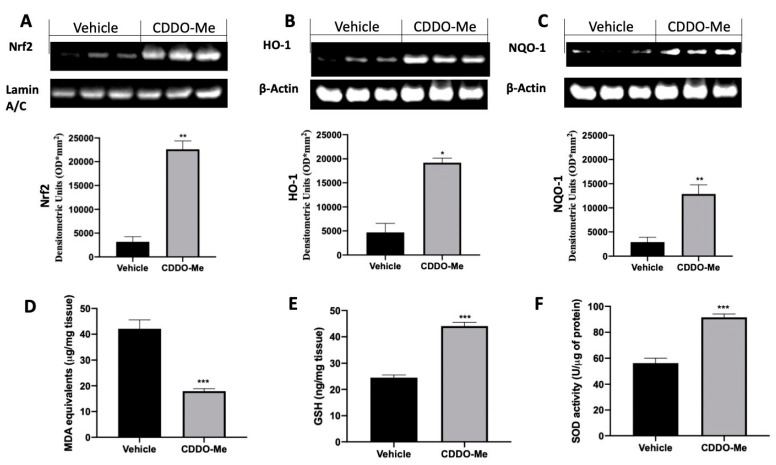
Administration of **2**-cyano-3,12-dioxooleana-1,9-dien-28-oic acid (CDDO-Me) reduced prooxidative alterations in endometrial explants: Western blot analysis of NRF2 (**A**), HO-1 (**B**), and NQO-1 (**C**)**;** malondialdehyde (MDA) levels (**D**), glutathione (GSH) levels (**E**), superoxide dismutase (SOD) activity (**F**). All values are expressed as mean ± standard error of the mean (SEM) of N observations. For in vivo studies, N represents the number of animals used. A *p*-value of less than 0.05 was considered significant. * *p* < 0.05 vs. vehicle, ** *p* < 0.01 vs. vehicle, *** *p* < 0.001 vs. vehicle.

In order to evaluate the effect of CCDO-Me on the oxidative balance, the Nrf2 pathway were examined. Western blot analysis revealed increased Nrf2 expression into the nucleus in tissues harvested from CDDO-Me-treated rats as compared to rats treated with vehicle (Figure 1A). Additionally, CDDO-Me treatment increased the expression of heme oxygenase 1 (HO-1) (Figure 1B) and NAD(P)H:quinone oxidoreductase-1 (NQO-1) (Figure 1C). Endometriotic explants from vehicle-treated rats showed elevated lipid peroxidation (Figure 1D) and low glutathione (GSH) levels (Figure 1E) and superoxide dismutase (SOD) activity (Figure 1F). CDDO-Me administration reduced MDA levels (Figure 1D) and increased GSH levels (Figure 1E) and SOD activity (Figure 1F).

### 2.2. Effect of CDDO-Me Treatment on the Inflammatory Mincroenvironment

**Figure 2:**
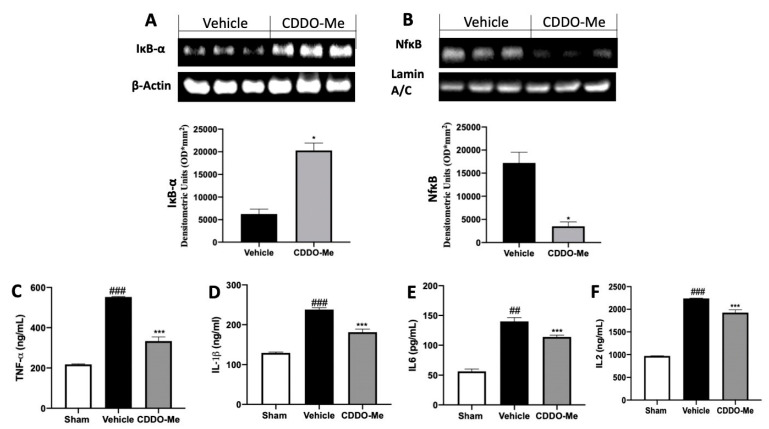
CDDO-Me administration reduced cytokines expressions: Western blot analysis of Ikb-α (**A**), NFkB (**B**); levels of tumor necrosis factor (TNF)-α (**C**), interleukin (IL)-1β (**D**), IL6 (**E**), IL2 (**F**). All values are expressed as mean ± standard error of the mean (SEM) of N observations. For in vivo studies, N represents the number of animals used. A *p*-value of less than 0.05 was considered significant. * *p* < 0.05 vs. vehicle, *** *p* < 0.001 vs. vehicle, ## *p* < 0.01 vs. sham, ### *p* < 0.001 vs. sham.

To test the anti-inflammatory activity of CDDO-Me administration on animals with endometriosis, the NFkB pathway was examined. Western blot analysis of endometrial explants from vehicle-treated rats showed low Ikb-α expression in the cytosol and high NFkB levels in the nucleus, while tissues harvested from CDDO-Me-administered rats showed increased Ikb-α expression in the cytosol (Figure 2A) and reduced NFkB levels in the nucleus (Figure 2B). Numerous inflammation-related mediators including TNF-α, IL-1β, IL6, and IL2 were evaluated in peritoneal fluids. Increased levels of proinflammatory cytokines were detected in samples from vehicle-treated rats, as compared to sham-treated animals. The levels of TNF-α (Figure 2C), IL-1β (Figure 2D), IL6 (Figure 2D), and IL2 (Figure 2F) in the peritoneal fluid collected from CDDO-Me-treated rats were significantly decreased.

### 2.3. Effect of CDDO-Me Treatment on COX-2 Expression and Apoptosis

**Figure 3:**
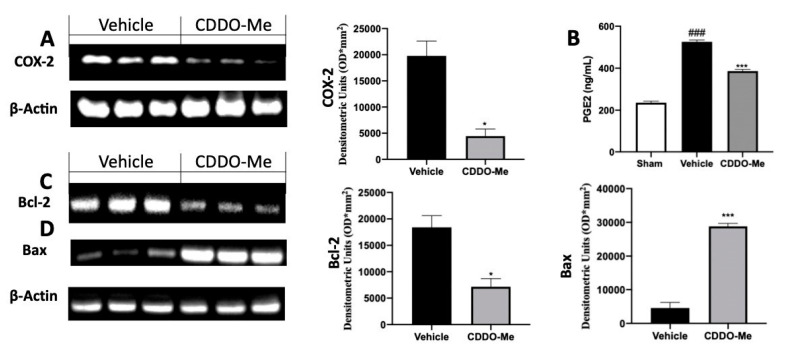
CDDO-Me administration reduced cyclooxygenase-2 (COX-2), prostaglandin E2 (PGE2), B-cell lymphoma 2 (Bcl-2), and B-cell lymphoma-associated X (Bax) expression: Western blot analysis of COX-2 (**A**), PGE2 levels (**B**); Western blot analysis of Bcl-2 (**C**), Bax (**D**). All values are expressed as mean ± standard error of the mean (SEM) of N observations. For in vivo studies, N represents the number of animals used. A *p*-value of less than 0.05 was considered significant. * *p* < 0.05 vs. vehicle, *** *p* < 0.001 vs. vehicle, ### *p* < 0.001 vs. sham.

Western blot analysis conducted on endometriotic loci from vehicle-treated rats showed increased COX-2 expression (Figure 3A). Additionally, the peritoneal fluids from vehicle-treated rats displayed increased PGE2 levels compared to the sham-treated animals (Figure 3B). CDDO-Me administration reduced COX-2 and PGE2 increased expression as compared to vehicle-treated rats (Figure 3A,B). Up-regulated PGE2 levels lead to the inhibition of the apoptotic pathway [24]. In order to investigate the effect of CDDO-Me administration on apoptosis, Western blot analysis was conducted. Tissues collected from vehicle-treated rats showed increased B-cell lymphoma 2 (Bcl-2) (Figure 3C) and decreased B-cell lymphoma-associated X (Bax) (Figure 3D) expression. CDDO-Me induced apoptosis, reducing Bcl-2 and increasing Bax levels (Figure 3C,D).

### 2.4. Effect of CDDO-Me Treatment on Angiogenesis

**Figure 4:**
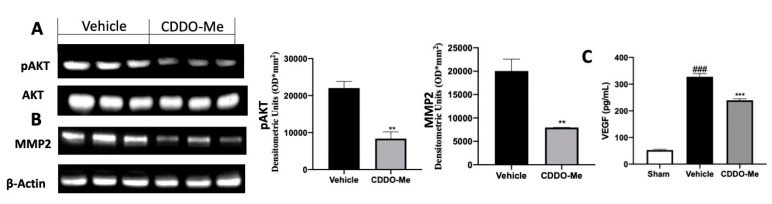
CDDO-Me administration reduced new vascular system formation: Western blot analysis of pAKT(**A**), matrix metalloproteinase-2 (MMP2 (**B**), VEGF levels (**C**). All values are expressed as mean ± standard error of the mean (SEM) of N observations. For in vivo studies, N represents the number of animals used. A *p*-value of less than 0.05 was considered significant. ** *p* < 0.01 vs. vehicle, *** *p* < 0.001 vs. vehicle, ### *p* < 0.001 vs. sham.

Several papers demonstrated the importance of a new vascular system formation in endometriosis [15]. Western blot analysis showed increased protein kinase B (AKT) phosphorylation in tissues harvested from vehicle-treated rats (Figure 4A), which was accompanied by increased matrix metalloproteinase-2 (MMP2) expression (Figure 4B) and vascular endothelial growth factor (VEGF) levels in the peritoneal fluids (Figure 4C). CDDO-Me administration reduced AKT phosphorylation Figure 4A) and in turn reduced MMP2 expression (Figure 4B) and VEGF levels (Figure 4C).

### 2.5. Macroscopic Analysis of the Effect of CDDO-Me Treatment on Endometriotic Foci

**Figure 5:**
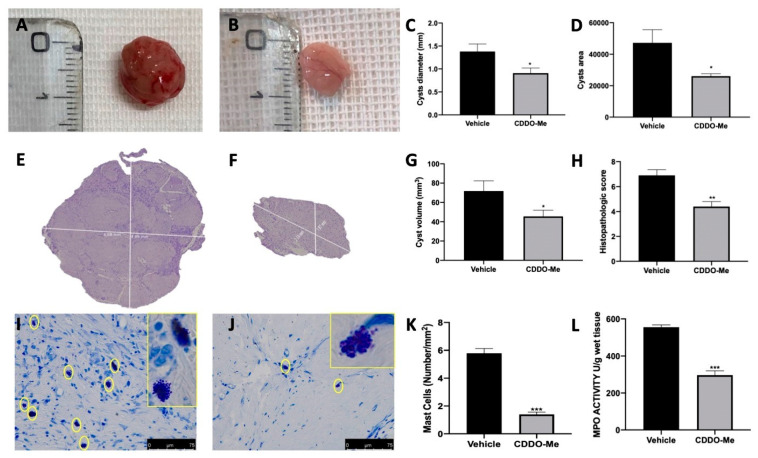
CDDO-Me administration reduced endometriotic foci. Macroscopic analysis: vehicle (**A**), CDDO-Me (**B**), cysts diameter (**C**), cysts area (**D**); histological analysis: vehicle (**E**), CDDO-Me (**F**), cysts volume (**G**); histopathological score (**H**). Toluidine blue staining: vehicle (**I**), CDDO-Me (**J**), mast cells number (**K**), myeloperoxidase (MPO) activity (**L**). All values are expressed as mean ± standard error of the mean (SEM) of N observations. For in vivo, studies N represents the number of animals used. A *p*-value of less than 0.05 was considered significant. * *p* < 0.05 vs. vehicle, ** *p* < 0.01 vs. vehicle, *** *p* < 0.001 vs. vehicle.

All animals from vehicle and CDDO-Me groups showed endometriosis implants, while sham-treated animals did not show any endometriosis lesions. The two groups did not show any different number of cysts. However, cysts diameter (Figure 5C), area (Figure 5D), and volume (Figure 5G) were smaller in the CDDO-Me-treated group (Figure 5B) compared to the vehicle-treated animals (Figure 5A). Histologically, endometriotic foci from vehicle-treated animals showed endometrial-type glands and stromal structure (Figure 5E,H). CDDO-Me administration reduced the histopathological signs of endometriosis (Figure 5F,H). Explants from vehicle-treated rats displayed increased mast cells recruitment (Figure 5I,K), while animals treated with CDDO-Me showed reduced mast cells infiltration into the lesions (Figure 5J,K). Additionally, CDDO-Me reduced myeloperoxidase (MPO) activity as compared to the vehicle-treated rats (Figure 5L).

### 2.6. Effect of CDDO-Me Treatment on Fibrosis Associated with Endometriosis

**Figure 6:**
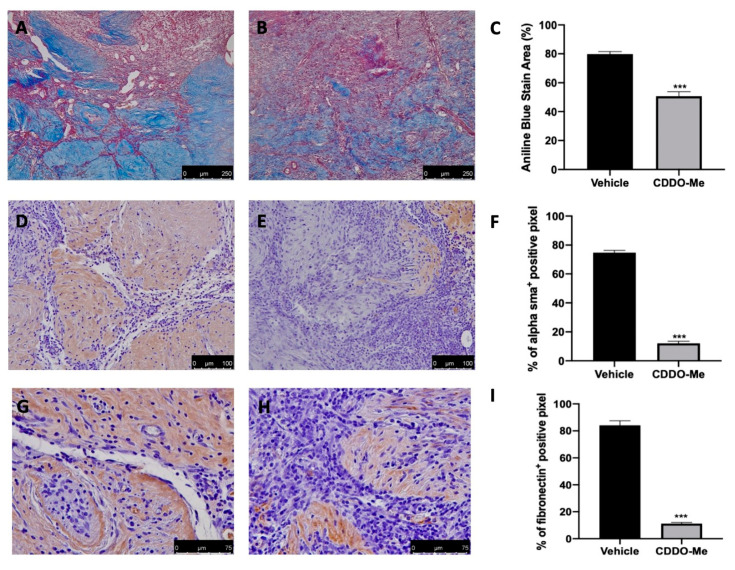
CDDO-Me administration reduced fibrosis. Masson trichrome staining: vehicle (**A**), CDDO-Me (**B**), aniline blue-stained Area (**C**); immunohistochemical analysis of α-smooth muscle actin (α-SMA): vehicle (**D**), CDDO-Me (**E**); graphical quantification of α-sma expression (**F**), immunohistochemical analysis of fibronectin: vehicle (**G**), CDDO-Me (**H**); graphical quantification of fibronectin expression (**I**). All values are expressed as mean ± standard error of the mean (SEM) of N observations. For in vivo studies, N represents the number of animals used. A *p*-value of less than 0.05 was considered significant. *** *p* < 0.001 vs. vehicle.

The presence of fibrosis was investigated by α-smooth muscle actin (α-SMA) and fibronectin immunolocalization and Masson’s trichrome staining. CDDO-Me administration significantly reduced the aniline blue-stained area (Figure 6B,C) as compared to vehicle-treated animals (Figure 6A,C). Immunohistochemical analysis was performed for α-SMA and fibronectin expression. Animals treated with CDDO-Me showed reduced α-SMA (Figure 6E,F) and fibronectin (Figure 6H,I) tissue expression compared to vehicle-treated rats (Figure 6D,F,G,I).

## 3. Discussion

Nowadays, endometriosis is described as an inflammatory and oxidative-like phenomenon.

Oxidative stress, defined as an imbalance between the levels of reactive oxygen species and those of antioxidants, plays a key role in endometriosis pathogenesis, resulting in peritoneal cavity inflammatory responses. Furthermore, the unbalanced oxidative/antioxidative status is responsible for increased adhesion and growth of endometrial lesions [25]. Endometriotic cells showed upregulated endogenous oxidative stress, with alteration of the ROS detoxification pathways [26]. Thus, modulating intracellular ROS signaling and the related inflammatory peritoneal microenvironment may be a therapeutic strategy for endometriosis. Several in vivo [27,28] and in vitro [29,30] studies reported that CDDO-Me is a potent regulator of the cellular antioxidant response and an inhibitor of inflammation.

In this paper, we show how CDDO-Me administration restored the oxidative and inflammatory peritoneal microenvironment during endometriosis, leading to a reduction of the lesions’ size.

The Nrf2 regulatory pathway has a key role in protecting cells from oxidative stress. By controlling the transactivation of several cytoprotective genes, Nrf2 is involved in many human diseases including endometriosis. A controlled activation of Nrf2 reduces the risk of endometriosis development by scavenging ROS and preventing DNA instability. In physiological conditions, it binds to Keap1 which is responsible for Nrf2 poly-ubiquitination by the CUL3–KEAP1 E1 ubiquitin ligase complex and degradation by the proteasome [31]. Increased oxidative stress modifies the reactive thiols of KEAP1 leading to Nrf2 accumulation into the cytosol caused by its decreased affinity for the ubiquitin ligase complex. Thus, Nrf2 translocates into the nucleus, heterodimerizes with other proteins, and then binds to antioxidant response elements (ARE), leading to the expression of many cytoprotective proteins with detoxifying and antioxidant roles, such as NQO-1 and HO-1 [32].

The activity of antioxidant systems is compromised in endometriosis patients. In comparison with controls, higher MDA, NO, and ROS levels were found in eutopic endometrium biopsies and lesions. After CDDO-Me treatment, Nrf2 translocation into the nucleus was increased, and subsequently increased HO-1 and NQO-1 expression was found. Additionally, CDDO-Me reduced lipid peroxidation and restored the cellular antioxidant system by increasing glutathione peroxidase and SOD activity. ROS generation leads to enhanced NFkB translocation, resulting in elevated angiogenic and proinflammatory mediators in endometriosis patients as compared to healthy subjects [33]. NFkB is involved in many pathways modulating cell survival, proliferation, apoptosis, neo-vascularization, adhesion, and invasion. In normal conditions, inactive NFkB complexes are located into the cytoplasm. NFkB dimers form a complex with the Ikb inhibitors, so NFkB is unable to bind to DNA. In response to inflammatory stimuli, Ikb inhibitory proteins are phosphorylated and degraded, leading to the activation of the cytoplasmic NFkB [34,35], which translocates into the nucleus for regulating pro-inflammatory gene expression [36]. In particular, the activation of the NFkB pathway is responsible for the increased levels of IL6, TNF-α, IL-1β, and IL2 in the peritoneal fluid. These cytokines have an important role in constructing the peritoneal environment that induces endometriosis progression [37,38]. The expression of these pro-inflammatory mediators further promotes NFkB activation [39,40]. During the late secretory phase, NFkB dysfunction was observed in endometriosis patients. Ikb kinase was downregulated coincidently with the reduction of NFkB DNA binding. Well in line with these clinical data, in our experimental model, increased oxidative stress induced the activation of the NFkB pathway and the increased the levels of proinflammatory mediators in the peritoneal fluid. CDDO-Me, by inhibiting Ikb-α degradation and reducing NFkB expression into the nucleus, was able to reduce the expression of pro-inflammatory mediators in the peritoneal fluid. The activation of the NFkB pathway by ROS also promotes invasion and proliferation of endometriotic cells by the increase of COX-2 and PGE2 expression [14,41]. COX-2 expression is responsible for the activation of multiple transcriptional pathways. It has been demonstrated that COX-2 expression was significantly elevated in endometriosis patients as compared to the healthy individuals [42]. Additionally, COX-2-induced PGE2 is an important antiapoptotic mediator that inhibits programmed cell death, which is one of the main characteristics of endometriosis. Increased PGE2 signaling is associated with increased levels of anti-apoptotic Bcl-2 and reduced expression of pro-apoptotic Bax proteins [24,43]. CDDO-Me administration by reducing COX-2 expression into endometriosis lesions and PGE2 levels in peritoneal fluids induced apoptosis. The literature revealed that in endometriosis patients, PGE2 expression is also associated with upregulated angiogenesis [44]. Angiogenesis is a mechanism in which the cellular matrix, proteolytic enzymes, and cytokines interact to create a new blood vascular system that evolves from the existing one.

This event has a key role in endometriosis development, due to the dependence of this pathology on the formation of a new vascular system. It has been described that PGE2 expression is involved in elevating MMP-2 and VEGF activity, inducing the AKT phosphorylation [15]. Our study showed that CDDO-Me, by reducing COX-2 expression and PGE2 levels, led to reduced AKT phosphorylation and MMP-2 and VEGF overexpression. A macroscopical analysis confirmed that CDDO-Me, by reducing oxidative stress and the inflammatory microenvironment, increasing apoptosis, and reducing vascular system formation, decreased diameter, area, and volume of the implants. Additionally, CDDO-Me reduced mast cell infiltration and neutrophil activation into the lesions. From the histological point of view, endometriosis loci are characterized by increased fibroblast-to-myofibroblast transdifferentiation (FMT) and epithelial–mesenchymal transition (EMT), resulting in augmented collagen production and cellular contractility, ultimately leading to fibrosis [45,46]. CDDO-Me was also able to reduce the expression of fibrotic proteins, including α-SMA, collagen and fibronectin, and leading to the decrease of fibrosis, extremely increased in ectopic endometria loci.

The current research has some limitations. The employed endometriosis model was applied by transplanting normal rat uterine tissue into the abdominal cavity of another rat. It does not accurately represent the pathogenesis of human endometriosis. However, rat models have a long history of being widely used in endometriosis research and have also been validated as a model that depicts the pathology dynamics.

In conclusion, our data suggest that CDDO-Me administration, by reducing oxidative stress and inflammation, would be useful for counteracting endometriosis grow and development.

## 4. Materials and Methods

### 4.1. Animals

Female Sprague–Dawley rats (Envigo, Milan, Italy) were used in this research. The University of Messina Review Board for animal care (OPBA) approved the study. All animal experiments agreed with the new Italian regulations, EU regulations, and the ARRIVE guidelines.

### 4.2. Experimental Protocol

Animals were randomly divided into two groups, donor or recipient, and endometriosis was established as already described [47]. To stimulate similar estrogen levels, donor rats were intraperitoneally injected with 10 IU of pregnant mare serum gonadotropin (PMSG, R&D System RP1782725000) and euthanized 41 h later by CO_2_ asphyxia. The uterus was removed and minced with scissors. Tissue from all donors was pooled, and the recipient animals were injected intraperitoneally with the equivalent of tissue from one uterus in 500 μL of PBS (ThermoFisher 10010023) along the midventral line. Endometriosis was allowed to develop for 7 days.

### 4.3. Experimental Groups

Rats were randomized and assigned to the following groups:

(1) Vehicle group: rats were subjected to experimental endometriosis as described above, and vehicle (0.1% dimethyl sulfoxide solvent (DMSO) (Merk 472301)) was intraperitoneally administered, on the 7th day and for the next 7 days.
(2) CDDO-Me group: rats were subjected to experimental endometriosis as described above, and CDDO-Me (5 mg/Kg) was intraperitoneally administered, on the 7th day and for the next 7 days.
(3) Sham group: rats were injected intraperitoneally with 500 μL of PBS without endometrial tissue, and vehicle (0.1% DMSO) was intraperitoneally administered, on the 7th day and for the next 7 days.

The dose of CDDO-Me was based on previous experiments [48,49]. Rats were sacrificed 14 days after endometriosis induction [47]. Peritoneal fluid and endometriotic implants were collected. Implants were excised from both groups, measured [50,51], and processed for histological and biochemical studies.

### 4.4. Determination of Reduced Glutathione Levels

The levels of reduced GSH were determined in endometriosis lesions and hippocampi to evaluate the endogenous antioxidant defenses. GSH levels were determined using a microplate reader at 412 nm and expressed as ng/mg wet tissue [52,53].

### 4.5. Measurement of Lipid Peroxidation

Lipoperoxidation was estimated in endometriosis lesions and hippocampi using the thiobarbituric acid reactive substances (TBARS) test [54,55]. The levels of MDA were determined using a microplate reader at 535 nm and expressed as μmol/mg of swet tissue.

### 4.6. Measurement of SOD Activity

In endometriosis lesions and hippocampi, determination of SOD activity was performed according to a previously described method [56,57,58]. SOD activity (U/μg protein) was determined using a microplate reader at 560 nm.

### 4.7. Analysis of MPO Activity

Myeloperoxidase activity with 3,30,5,50-tetramethylbenzidine (TMB) was measured in endometriosis lesions and hippocampi as already described [59,60]. Absorbance was measured at 450 nm to estimate MPO activity.

### 4.8. Enzyme-Linked Immunosorbent Assay

Peritoneal fluid was collected. VEGF, IL6, TNF-α, IL-1β, IL2, and PGE2 were determined using an ELISA kit (BioLegend, San Diego, CA, USA) [61,62].

### 4.9. Histological Examination

For histopathological investigations, endometriosis lesions were fixed at room temperature in a buffered formaldehyde solution (10% in PBS); histological sections were stained with hematoxylin and eosin (H&E) and evaluated using a Leica DM6 microscope (Leica Microsystems SpA, Milan, Italy) equipped with a motorized stage and associated with Leica LAS X Navigator software (Leica Microsystems SpA, Milan, Italy) [63]. Histopathologic scores were evaluated as described previously [63]. Additionally, lesion volume was calculated according to the formula: V = (length × width^2^) × 0.5 [64]. The degree of fibrosis was evaluated by the Masson trichrome staining performed according to the manufacturer’s protocol (Bio-Optica, Milan, Italy) [65,66]. Mast cells evaluation was performed by toluidine blue staining [67].

### 4.10. Immunohistochemical Analysis

Immunohistochemical localization of α-SMA and fibronectin was performed in endometriosis lesions as already described [68,69]. The sections were incubated overnight with primary antibodies: anti-α-SMA antibody (Santa Cruz Biotechnology, CGA7) or anti-fibronectin antibody (Santa Cruz Biotechnology, sc-271098). All sections were washed with PBS and then treated as previously reported [70,71]. The stained sections were observed using a Leica DM6 microscope following a typical procedure [72]. The histogram profile is related to the positive pixel intensity value obtained [73].

### 4.11. Western Blot Analysis

Western blots were performed as already described [74,75]. Specific primary antibody: each antibody, anti-Nrf2 (Santa Cruz Biotechnology, sc-365949), anti-HO-1 (Santa Cruz Biotechnology, sc-390991), anti-NQO-1 (Santa Cruz Biotechnology, sc-32793), anti-IkB-α (Santa Cruz Biotechnology, sc-1643), anti-Nf-kb (Santa Cruz Biotechnology, sc-8008), anti-COX-2 (Santa Cruz Biotechnology, sc-376861), anti-Bcl-2 (Santa Cruz Biotechnology, sc-7382), anti-Bax (Santa Cruz Biotechnology, sc-7480), anti-MMP2 (Santa Cruz Biotechnology, sc-13594), anti-*p*-AKT (Abcam), anti-AKT (Abcam), was mixed in a 5% *w*/*v* of non-fat dried milk solution and incubated at 4 °C, overnight. Afterwards, blots were incubated with peroxidase-conjugated bovine anti-mouse IgG secondary antibody or peroxidase-conjugated goat anti-rabbit IgG (Jackson Immuno Research) for 1 h at room temperature. To verify the equal amounts of protein, membranes were also incubated with an antibody against β-actin (Santa Cruz Biotechnology). Signals were detected with an enhanced chemiluminescence detection system reagent (Super-Signal West Pico Chemiluminescent Substrate, Pierce). The relative expression of the protein bands was quantified by densitometry with Bio-Rad ChemiDoc XRS software and standardized to β-actin levels [76]. Images of blot signals were imported to analysis software (Image Quant TL, v2003).

### 4.12. Statistical Evaluation

All values are expressed as mean ± standard error of the mean (SEM) of N observations. For in vivo studies, N represents the number of animals used. The results were analyzed by *t*-test, and the Kolmogorov–Smirnov test was applied to analyze the normal distribution of the data (Prism 8 for macOS version 8.2.1 (279)). A *p*-value of less than 0.05 was considered significant; * *p* < 0.05 vs. vehicle, ** *p* < 0.01 vs. vehicle, *** *p* < 0.001 vs. vehicle, ^#^ *p* < 0.05 vs. sham, ^##^ *p* < 0.01 vs. sham, ^###^ *p* < 0.001 vs. sham.

## Data Availability

The data presented in this study are available on request from the corresponding author.
